# Quantifying inbreeding avoidance through extra-pair reproduction

**DOI:** 10.1111/evo.12557

**Published:** 2014-12-03

**Authors:** Jane M Reid, Peter Arcese, Lukas F Keller, Ryan R Germain, A Bradley Duthie, Sylvain Losdat, Matthew E Wolak, Pirmin Nietlisbach

**Affiliations:** 1 School of Biological Sciences, Institute of Biological and Environmental Sciences, Zoology Building, University of AberdeenTillydrone Avenue, Aberdeen, AB24 2TZ, Scotland; 3 Department of Forest and Conservation Sciences, University of British ColumbiaVancouver, BC, V6T 1Z4, Canada; 4 Institute of Evolutionary Biology and Environmental Studies, University of ZurichWinterthurerstrasse 190, 8057, Zurich, Switzerland

**Keywords:** Inbreeding depression, kinship, mate choice, paternity, polyandry, relatedness

## Abstract

Extra-pair reproduction is widely hypothesized to allow females to avoid inbreeding with related socially paired males. Consequently, numerous field studies have tested the key predictions that extra-pair offspring are less inbred than females’ alternative within-pair offspring, and that the probability of extra-pair reproduction increases with a female's relatedness to her socially paired male. However, such studies rarely measure inbreeding or relatedness sufficiently precisely to detect subtle effects, or consider biases stemming from failure to observe inbred offspring that die during early development. Analyses of multigenerational song sparrow (*Melospiza melodia*) pedigree data showed that most females had opportunity to increase or decrease the coefficient of inbreeding of their offspring through extra-pair reproduction with neighboring males. In practice, observed extra-pair offspring had lower inbreeding coefficients than females’ within-pair offspring on average, while the probability of extra-pair reproduction increased substantially with the coefficient of kinship between a female and her socially paired male. However, simulations showed that such effects could simply reflect bias stemming from inbreeding depression in early offspring survival. The null hypothesis that extra-pair reproduction is random with respect to kinship therefore cannot be definitively rejected in song sparrows, and existing general evidence that females avoid inbreeding through extra-pair reproduction requires reevaluation given such biases.

Extra-pair reproduction, and polyandry more generally, are widely hypothesized to have evolved to allow females to avoid inbreeding with related socially paired or previously mated males, thereby circumventing constraints on initial mate choice (Jennions and Petrie 2000; Tregenza and Wedell 2000; Griffith et al. 2002; Kempenaers 2007; Firman and Simmons 2008; Griffith and Immler 2009). Such hypotheses stem from the observation that inbreeding often depresses offspring fitness (Lynch and Walsh 1998; Charlesworth and Charlesworth 1999; Keller and Waller 2002), creating widely–held expectations that there will inevitably be selection against inbreeding (Tregenza and Wedell 2000; Szulkin et al. 2013). Because random extra-pair reproduction is not necessarily expected to change the relatedness between a female and the sire of her offspring on average, some form of nonrandom inbreeding avoidance or preference through extra-pair reproduction is expected to be required to alter the mean inbreeding level of a female's offspring and thereby alter her genetic contribution to subsequent generations (Szulkin et al. 2013; but see Hosken and Blanckenhorn 1999).

Accordingly, numerous field studies have attempted to test three key predictions: first, that socially paired females do systematically alter their relatedness to the sire of their offspring through extra-pair reproduction; second, that observed changes in relatedness differ from those expected under some null model of random extra-pair reproduction and therefore constitute active inbreeding avoidance or preference defined as negative or positive deviations from null expectation; and third, that the probability of extra-pair reproduction varies with a female's relatedness to her socially paired male (e.g., Blomqvist et al. 2002; Foerster et al. 2003; Tarvin et al. 2005; Suter et al. 2007; Cohas et al. 2008; Brouwer et al. 2011; Wang and Lu 2011; Varian-Ramos and Webster 2012; Harrison et al. 2013; Kingma et al. 2013; Leclaire et al. 2013). Such studies report diverse effects, including apparent inbreeding avoidance (e.g., Blomqvist et al. 2002; Foerster et al. 2003; Brouwer et al. 2011), preference (e.g., Wang and Lu 2011), and tolerance (i.e., random extra-pair reproduction with respect to relatedness, e.g., Kingma et al. 2013; Leclaire et al. 2013), and no overarching patterns are yet evident (Akçay and Roughgarden 2007; Kempenaers 2007; Szulkin et al. 2013). Meanwhile, the probability of extra-pair reproduction commonly increases with a female's relatedness to her socially paired male (e.g., Blomqvist et al. 2002; Tarvin et al. 2005; Cohas et al. 2008; Brouwer et al. 2011; Varian-Ramos and Webster 2012; Kingma et al. 2013; Leclaire et al. 2013), but does not always do so (Kempenaers 2007). These diverse results might indicate that relationships between extra-pair reproduction, relatedness and inbreeding vary among systems, potentially reflecting variation in population ecology or life-history and associated constraints on mate choice (Keller and Arcese 1998; Jennions and Petrie 2000; Jamieson et al. 2009; Kingma et al. 2013). However, apparent diversity might also arise because such relationships are extremely challenging to quantify in wild populations, meaning that estimates might be subject to substantial sampling variance and divergent bias.

## MEASURING RELATEDNESS AND INBREEDING

Key prerequisites to understanding patterns of extra-pair reproduction in relation to inbreeding are to adequately measure the relatedness or kinship between a female and her socially paired male and her actual and potential extra-pair males, and/or to quantify the corresponding inbreeding levels of actual and potential offspring (Szulkin et al. 2013). The coefficient of kinship (*k*) between any female–male pairing can be calculated from pedigree data, where *k* equals the coefficient of inbreeding (*f*) of resulting offspring and quantifies the probability that two homologous alleles will be identical by descent relative to the defined pedigree baseline (thereby quantifying expected identity by descent, Keller and Waller 2002; Slate et al. 2004). However, pedigree estimates of *k* and *f* are prone to error and bias when individuals have unknown or misassigned parents, including misassigned sires due to unobserved extra-pair paternity (Keller et al. 2001; Ewing et al. 2008; Reid et al. 2014). If extra-pair paternity were nonrandom with respect to relatedness, as is widely hypothesized, then tests of hypotheses relating female extra-pair reproduction to *k*, *f*, and fitness might be biased. Furthermore, the precision with which pedigree analyses can quantify variation in *k* and *f* increases with pedigree depth and completeness. Detecting small but potentially evolutionarily important degrees of inbreeding and inbreeding avoidance or preference in relation to expected identity by descent will therefore require complete, error-free pedigrees that span multiple generations of ancestors of focal females and their actual and potential social and extra-pair males (Szulkin et al. 2013). Such data are rarely available, not least because extra-pair reproduction and other forms of polygynandry impede construction of accurate pedigrees based on social pairings and matings observed during long-term field studies (e.g., Keller et al. 2001; Brommer et al. 2007; Szulkin et al. 2007; Reid et al. 2014).

The challenges of compiling adequate pedigrees mean that field studies relating extra-pair reproduction to relatedness and inbreeding have almost exclusively used metrics of genotypic similarity or heterozygosity computed across small sets of molecular markers (e.g., Blomqvist et al. 2002; Foerster et al. 2003; Tarvin et al. 2005; Suter et al. 2007; Cohas et al. 2008; Brouwer et al. 2011; Wang and Lu 2011; Varian-Ramos and Webster 2012; Harrison et al. 2013; Kingma et al. 2013; Leclaire et al. 2013). Although molecular metrics can capture realized rather than expected identity by descent (Forstmeier et al. 2012), metrics calculated from few markers are subject to substantial sampling variance that might typically swamp the variation in identity by descent arising from the degree of inbreeding occurring in wild populations with biparental sexual reproduction. Such metrics might consequently be only weakly correlated with *f*, except in populations whose substructures or mating systems create unusually large variances (Balloux et al. 2004; Slate et al. 2004; Robinson et al. 2013). They might also be weakly correlated with realized rather than expected identity by descent, except in species with few linkage groups and infrequent recombination and correspondingly high linkage disequilibria (Forstmeier et al. 2012). Estimates of relatedness or relationship between specific individuals, or inbreeding levels of resulting offspring, derived from few markers can consequently be very imprecise (Balloux et al. 2004). First- or second-order relatives might be distinguishable from unrelated individuals with some confidence, potentially allowing detection of extra-pair reproduction that exchanges first- or second-order relatives for unrelated mates or vice versa. However, even such categorical assignments can be uncertain with frequent misclassification (Csilléry et al. 2006; Santure et al. 2010). Meanwhile, extra-pair reproduction that causes much more subtle changes in offspring inbreeding level might have nontrivial fitness consequences if inbreeding depression in offspring fitness is substantial. Studies that estimate relatedness or inbreeding from sparse genotypes, or estimate *k* and *f* from shallow, incomplete, or inaccurate pedigrees, are unlikely to reliably detect such strategies (Smith et al. 2005; Csilléry et al. 2006).

## OBSERVATION BIAS

A further major difficulty is that estimated relationships between extra-pair reproduction, inbreeding and relatedness could be biased by failure to observe offspring that die before DNA can be sampled (typically sometime postbirth or posthatch) and hence before paternity can be assigned or *f*, *k*, relatedness, or heterozygosity estimated. Specifically, if offspring survival to sampling were correlated with inbreeding (i.e., there was inbreeding depression in early survival), and therefore also correlated with the relatedness between a female and the sire of her offspring, then estimates of the degrees to which females undertake extra-pair reproduction in relation to relatedness, or alter offspring inbreeding level through that extra-pair reproduction, could be biased. Such biases would arise if inbred within-pair offspring produced by closely related social pairings were more likely to die before observation than relatively outbred within-pair offspring produced by less closely related social pairings. The proportion of offspring sired by a female's socially paired male could then be underestimated to a degree that depends on the relatedness between the female and her socially paired male. Furthermore, inbred extra-pair offspring sired by females’ relatives might be more likely to die before being observed than outbred extra-pair offspring sired by nonrelatives, causing the mean reduction in offspring inbreeding level accrued through extra-pair reproduction to be overestimated. Analogous observation failure has been shown to bias inference of male fertilization success from subsequently observed paternity, and of relationships between fertilization success and offspring viability (Olsson et al. 1999; Bretman et al. 2004; García-González 2008). However, such biases have not generally been explicitly considered by studies relating extra-pair reproduction to inbreeding and relatedness, even though inbreeding depression in early survival is commonplace and can be severe (Lynch and Walsh 1998; Olsson et al. 1999; Keller and Waller 2002; Kempenaers 2007; Hemmings et al. 2012).

Understanding the magnitude and mechanisms of selection on female extra-pair reproduction therefore requires field studies that measure subtle variation in *k* or relatedness among focal females and their socially paired and actual and potential extra-pair males (or measure *f* or heterozygosity of resulting offspring) with high accuracy and precision, and that relate these variables to extra-pair reproduction while eliminating or quantifying bias due to failure to observe offspring that die early. Arguably, no such studies yet exist. Accordingly, we used comprehensive pedigree data from free-living song sparrows (*Melospiza melodia*) to quantify (1) whether females systematically altered their *k* with the sire of their offspring, and hence altered offspring *f*, through observed extra-pair reproduction; (2) whether the observed change in *k* differed from that expected given random extra-pair reproduction among females and their potential extra-pair males and therefore constituted nonrandom inbreeding avoidance or preference with respect to expected identity by descent; and (3) whether the probability of extra-pair reproduction varied with a female's *k* with her socially paired male. We then used simulations to quantify the magnitude of bias that could result from failure to observe inbred offspring that died early, and discuss the general implications for empirical estimates of inbreeding strategy.

## Materials and Methods

### STUDY SYSTEM AND PEDIGREE

The hypothesis that females systematically alter their relatedness to the sire of their offspring (and hence alter offspring inbreeding level) through extra-pair reproduction is most appropriately tested in socially monogamous populations where females encounter a diversity of close, distant and nonrelatives as potential social and extra-pair mates, and where such variation is likely to have existed across sufficient generations for associated inbreeding strategies to have evolved (Szulkin et al. 2013). One appropriate system is a song sparrow meta-population occupying island and mainland habitat patches in coastal British Columbia, Canada and Washington State, USA, where small, resident subpopulations are linked by dispersal (Smith et al. 1996; Keller and Arcese 1998; Wilson and Arcese 2008).

Mandarte island, BC, holds a song sparrow subpopulation that recently numbered 10–50 breeding pairs (Smith et al. 2006; Sardell et al. 2010). Each year since 1975, all nests were located, clutch sizes were recorded, and all offspring surviving to approximately six days posthatch were banded with unique combinations of metal and colored bands (Smith et al. 2006). The occasional immigrants to Mandarte (1.1 year^−1^ on average) were mist-netted and banded soon after arriving. All social pairings of adults, and hence the social parents of all offspring, were identified (Sardell et al. 2010; Reid et al. 2014). All territories occupied by social pairs, or by males that remained socially unpaired due to the typically male-biased adult sex ratio, were mapped by plotting song posts and boundary disputes (e.g., Arcese 1987, 1989; Smith et al. 2006; Akçay et al. 2011). Nonterritorial “floater” males were also identified (Arcese 1987; Sardell et al. 2010). Both sexes have median reproductive life spans of two years (interquartile range: one to four years). Females typically rear up to three broods of offspring per year with the same or different socially paired males.

To identify genetic parents and quantify extra-pair reproduction, 99.6% of adults and offspring banded during 1993–2012 were blood-sampled and initially genotyped at 13 highly polymorphic microsatellite loci. All genetic mothers matched those assigned from maternal behavior (Sardell et al. 2010). Bayesian full probability models assigned sires to 99.7% of sampled offspring with ≥95% individual-level confidence (Sardell et al. 2010; Reid et al. 2014). Assigned paternities were subsequently verified using up to 170 microsatellites, ensuring virtually complete confidence. Overall, about 28% of sampled offspring were assigned to males other than a female's socially paired male and hence identified as extra-pair offspring, and about 44% of broods contained ≥1 extra-pair offspring. However, offspring that died before blood-sampling at six days posthatch were not generally genotyped, meaning that their paternity was not verified (Taylor et al. 2010). Sexes of blood-sampled offspring were ascertained from their CHD-1 genotype (Postma et al. 2011).

The social parentage data were used to compile a pedigree spanning sparrows banded during 1975–2012. Genetic paternities were then used to correct pedigree error stemming from extra-pair reproduction during 1993–2012 (Reid et al. 2014). To minimize remaining pedigree error, sparrows hatched during 1991–1992 that bred subsequently were genotyped and their paternity was corrected as far as available samples allowed (Reid et al. 2014). The pedigree was therefore sufficiently deep, complete, and accurate to estimate *k* between contemporary females and their observed and potential socially paired and extra-pair males with high accuracy and precision (see Data Restriction).

### DIFFERENCE IN KINSHIP THROUGH OBSERVED EXTRA-PAIR REPRODUCTION

For each observed (i.e., blood-sampled at six days posthatch) extra-pair offspring, standard pedigree algorithms were used to calculate *k* between the female (i.e., the extra-pair offspring's mother) and her socially paired male (*k*_SOC_) and her extra-pair male (i.e., the sire of the extra-pair offspring, *k*_EP_). The difference in *k* between a female and her socially paired versus extra-pair male was calculated as *k*_DIFF_ = *k*_EP_ – *k*_SOC_. Negative and positive values of *k*_DIFF_ therefore indicate that a female reduced or increased her *k* with the sire of her offspring, and hence reduced or increased offspring *f*, through extra-pair reproduction. For reference, *k* = 0.25, 0.125, 0.0625, and 0, respectively, indicate full-sib, half-sib, first-cousin (or equivalent), and unrelated pairings among otherwise outbred individuals. Immigrants to Mandarte are assumed to be unrelated to existing residents upon arrival, defining *k* = 0 with their immediate mates (Reid et al. 2006, 2014). However, immigrants could subsequently inbreed with their own descendants, allowing *k*_SOC_ > 0, *k*_EP_ > 0, and *k*_DIFF_ ≠ 0. Immigration was sufficient to maintain substantial within-population variation in *k* and *f* (see Results).

A linear mixed model with Gaussian error structure and fixed effects of offspring sex was fitted to test whether mean *k*_DIFF_ differed from zero across females’ observed extra-pair offspring, thereby testing whether females systematically increased or decreased *k* with the sire of their offspring (and hence increased or decreased *f* of their sons and/or daughters) through extra-pair reproduction. Nested random brood, social pairing, and female effects were fitted to account for any correlations in *k*_DIFF_ across multiple extra-pair offspring observed in the same brood or produced by the same social pairing or female.

### DIFFERENCE IN KINSHIP THROUGH RANDOM EXTRA-PAIR REPRODUCTION

Assessing whether observed *k*_DIFF_ differed from that expected given random extra-pair reproduction requires each female's set of potential extra-pair males to be identified. Extra-pair paternity is highly spatially restricted in Mandarte's song sparrows (Sardell et al. 2010), as in other systems (e.g., Suter et al. 2007; Kingma et al. 2013). Specifically, approximately 89%, 8.5%, and 2.5% of observed extra-pair offspring were, respectively, sired by “first-neighbor” males that shared a territory boundary with the offspring's mother, by “second-neighbor” males that shared a territory boundary with a first-neighbor, and by “non-neighbor” males that occupied more distant territories or were nonterritorial floaters (updated from Sardell et al. 2010). The sets of first-, second-, and non-neighbor males pertaining to every breeding attempt made by every female were identified from territory maps. The distributions of *k* between the female that produced each observed extra-pair offspring and her concurrent socially paired and first-, second-, and non-neighbor males were computed, thereby quantifying each female's opportunity to alter her *k* with the sire of her offspring (and hence alter offspring *f*) through extra-pair reproduction.

To generate the null distribution of mean *k*_DIFF_ arising from random extra-pair reproduction, a single extra-pair male was assigned to each breeding attempt by sampling from the female's concurrent first-, second-, and non-neighbor males with probabilities 0.89, 0.085, and 0.025, respectively. For every observed extra-pair offspring, the difference in *k* between the female and her randomly assigned extra-pair male (*k*_EP.RAND_) versus her observed socially paired male was calculated as *k*_DIFF.RAND_ = *k*_EP.RAND_ – *k*_SOC_. Mean *k*_DIFF_ estimated across all observed extra-pair offspring was then compared to the distribution of mean *k*_DIFF.RAND_ generated across 10,000 randomizations. Conclusions remained similar when the probabilities of sampling extra-pair males from each female's first-, second-, and non-neighbors were substantially altered (see Results), when immigrants were excluded, and considering median rather than mean *k*_DIFF_.

### DATA RESTRICTION

Accurate estimation of *k*_DIFF_ and *k*_DIFF.RAND_ requires accurate estimation of *k*_SOC_, *k*_EP_, and *k*_EP.RAND_. This in turn requires sufficiently deep, accurate pedigree data for all three adults involved in each extra-pair offspring (i.e., the female, her socially paired male, and her observed or random extra-pair male). The full song sparrow pedigree spanning 1975–2012 presumably contains paternity error for individuals hatched during 1975–1992 due to unobserved extra-pair reproduction before 1993, causing downstream error in estimates of *k*_SOC_, *k*_EP_, and *k*_EP.RAND_ (Reid et al. 2014). To minimize such error, analyses were restricted to extra-pair offspring banded during 2007–2012. In these years, all ancestors of all adult song sparrows back to (and including) their great-grandparents were known and genetically verified, or were immigrants or their ancestors and hence defined as unrelated (Supporting Information). This restriction equates to extra-pair offspring whose actual and potential ancestors back to great-great-grandparents were all verified or defined as unrelated (Supporting Information). Although some great-great-great-grandfathers and more distant ancestors of these offspring will presumably still be misassigned, iterative pedigree correction across successive generations suggested that remaining error in *k*_SOC_, *k*_EP_, and *k*_DIFF_ is very small (Supporting Information). This restriction to offspring with completely verified great-great-grandparents is much stricter than commonly applied in wild population pedigree analyses, where offspring with assigned (but not necessarily genetically verified) grandparents are typically retained (e.g., Keller et al. 2001; Szulkin et al. 2007).

The coefficients *k* and *f* measure expected identity by descent rather than realized identity by descent resulting from shared ancestry and Mendelian segregation variance. However, in species with numerous linkage groups and frequent recombination, the absolute sampling deviation between *k* and realized identity by descent is expected to be small for distant outbred relatives (Hill and Weir 2011). The deviation is likely to be even smaller when these relatives are themselves somewhat inbred, because gametic variance is reduced. Variation in *k* and realized identity by descent will therefore be correlated.

### KINSHIP AND THE PROBABILITY OF EXTRA-PAIR REPRODUCTION

Binomial linear mixed models, with the numbers of banded extra-pair offspring and total banded offspring per brood as binomial numerator and denominator, respectively, and logit link function, were used to test whether the probability that a female's banded offspring was sired by an extra-pair male (i.e., was an extra-pair offspring) varied with her kinship with her socially paired male (*k*_SOC_), and to estimate the slope of the regression of the (logit) probability of extra-pair reproduction on *k*_SOC_ (β_EPR_).

To inspect the degree to which β_EPR_ estimated across banded offspring might potentially be biased by nonrandom offspring mortality prior to banding (and consequent failure to observe paternity), further mixed models were fitted to test whether total clutch size or brood size at banding (assuming Poisson error structures and log link function), or the probability that an offspring would die before banding (with the number of eggs that failed to produce a banded chick and total clutch size as binomial numerator and denominator and logit link function), varied with *k*_SOC_.

These models were fitted to individual broods where ≥1 offspring survived to banding and paternity assignment during 2007–2012, and hence where some degree of extra-pair and/or within-pair reproduction was observed. Fixed year effects and random female and social pairing effects were fitted to account for among-year variation and correlations among broods reared by individual females and social pairings. Results were quantitatively similar when Bayesian models were fitted, allowing explicit estimation of additive overdispersion. To visualize patterns of variation, mean clutch size and brood size, and the mean proportions of extra-pair offspring in each banded brood and of eggs that died before banding, were calculated across breeding attempts pooled into discrete categories of *k*_SOC_ (see Results).

### BIAS DUE TO NONRANDOM OFFSPRING MORTALITY

In common with all such studies, the preceding analyses of extra-pair reproduction only considered offspring that survived to posthatch DNA-sampling and paternity assignment, and ignored offspring from the same broods that died earlier and hence whose sire was unverified. However, if there was inbreeding depression in early offspring survival, then failure to observe paternity would depend on *k*_SOC_ and *k*_EP_. We used simulations to investigate the potential magnitude of consequent bias in estimates of *k*_DIFF_ and β_EPR_ given the song sparrow data structure.

For all individual eggs (i.e., assumed conceived offspring) in all clutches where ≥1 offspring survived to banding, the sire was randomly assigned as the female's observed socially paired male with probability 0.76 (thereby defining a within-pair offspring), or assigned as an extra-pair sire randomly sampled from the female's neighbors (thereby defining an extra-pair offspring, with a single extra-pair sire assigned per brood). This simulated extra-pair paternity rate of 24% matched the population-wide rate observed at banding during 2007–2012 (see Results). Values of *k*_SOC_ and *k*_EP_ were then calculated from the pedigree given each conceived offspring's observed socially paired parents and simulated sire. The mean simulated difference between *k*_SOC_ and *k*_EP_ (*k*_DIFF.ALL.SIM_ = *k*_EP_ – *k*_SOC_), and the regression of the (logit) probability that an offspring would be sired by an extra-pair male on *k*_SOC_ (β_EPR.ALL.SIM_) were then estimated across all simulated conceived offspring (i.e., with zero failure to observe the assigned paternity) using the same methods as for the real observed song sparrow offspring.

Each simulated offspring's probability of surviving to hypothetical banding and observation of genetic paternity was then calculated as *S*_p_ = exp(–δ*f* + η), where δ is a population-wide decrement due to inbreeding and η is an individual environmental deviation. Individuals with *S*_p_ values below the 17th percentile of the full distribution were defined as dead before banding so that the simulated egg to banding survival rate matched the 83% observed in the real dataset (see Results). The mean difference between *k*_SOC_ and *k*_EP_, and the regression of the (logit) probability that an offspring would be sired by an extra-pair male on *k*_SOC_ were then calculated across offspring that were simulated to survive to banding (*k*_DIFF.SURV.SIM_ and β_EPR.SURV.SIM_, respectively) as previously. The magnitude of bias in the estimated degree to which females altered their *k* to the sire of their offspring through extra-pair reproduction, and in the estimated relationship between extra-pair reproduction and *k*_SOC_, which resulted from simulated failure to observe offspring that died early, were then calculated as *k*_BIAS_ = *k*_DIFF.SURV.SIM_ – *k*_DIFF.ALL.SIM_ and β_BIAS_ = β_EPR.SURV.SIM_ – β_EPR.ALL.SIM_, respectively.

The simulation was repeated for 10,000 iterations. A value of δ was drawn from a uniform distribution with range 0–3 for each iteration, and η was drawn from a uniform distribution with range 0–Ɯ for every conceived offspring within each iteration, where Ɯ was itself drawn from a uniform distribution with range 0.25–2 for each iteration (thereby controlling the iteration-level magnitude of random environmental variation in *S*_p_). The realized magnitude of inbreeding depression in offspring survival to hypothetical banding (*B*_ID_) within each iteration was calculated in lethal equivalents as the slope of a regression of ln(*S*_o_) on *f*_o_ (i.e., ln(*S*_o_) = A – *B*_ID_ · *f*_o_), where *S*_o_ is the observed proportion of conceived offspring (i.e., eggs) that survived to hypothetical banding within each of 10 categories defined with respect to *f*, and *f*_o_ is mean *f* of all offspring within each category (Morton et al. 1956; Lynch and Walsh 1998). The defined categories of *f* contained approximately equal numbers of offspring. The defined ranges of δ and Ɯ created wide ranges of *B*_ID_, including *B*_ID_ = 0 (see Results). Conclusions remained similar when simulations were rerun after varying the number and distribution of unobserved offspring and the global rates of extra-pair paternity and offspring mortality both among and across years.

Analyses were run in R version 2.15.2 (R Development Core Team 2012) using packages kinship2, lme4, and nlme. Means are reported ±1 SD unless otherwise stated.

## Results

### DIFFERENCE IN KINSHIP THROUGH OBSERVED EXTRA-PAIR REPRODUCTION

**Figure 1:**
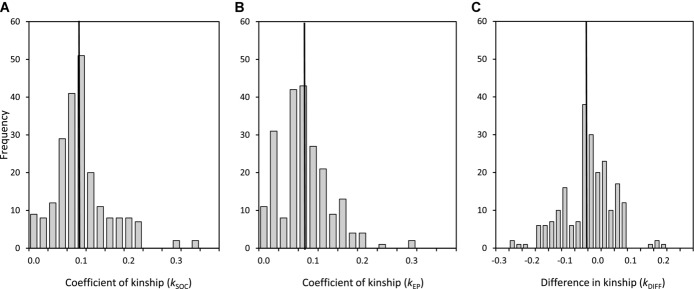
Distributions of the coefficient of kinship between a female song sparrow and her (A) socially paired male (*k*_SOC_) and (B) extra-pair male (*k*_EP_), and (C) the difference between the two (*k*_DIFF_ = *k*_EP_ – *k*_SOC_) across 216 observed extra-pair offspring. Vertical lines demarcate means.

During 2007–2012, there were 216 banded extra-pair offspring whose mother and her socially paired and extra-pair males all had genetically verified or immigrant ancestors back to great-grandparents (i.e., the offspring's great-great-grandparents). These 216 offspring represented 130 broods, and were produced by 60 females and 110 parent trios. Only five broods contained offspring of two extra-pair males. Across all 216 extra-pair offspring, mean *k*_SOC_ between the female and her socially paired male was 0.109 ± 0.058 (median 0.102, range 0.000–0.356, Fig. 1A), whereas mean *k*_EP_ between the female and her extra-pair male was 0.091 ± 0.053 (median 0.083, range 0.000–0.304, Fig. 1B). There was therefore substantial variation in potential and observed inbreeding through both within-pair and extra-pair reproduction.

The difference in *k* between a female and her socially paired versus extra-pair male (*k*_DIFF_) differed from zero for 211 of 216 extra-pair offspring (97.7%). Therefore, extra-pair reproduction almost always altered the *f* of a female's extra-pair offspring relative to her within-pair offspring. Raw mean *k*_DIFF_ across the 216 extra-pair offspring was –0.018 ± 0.077 (median –0.011, range –0.241–0.212, Fig. 1C), indicating that females slightly reduced offspring *f* through extra-pair reproduction on average. Although the model-predicted reduction was small (–0.020 ± 0.009 SE), it differed from zero (*t* = –2.3, *P* = 0.027). However, *k*_DIFF_ was negative in only 125 (57.9%) of 216 cases and showed considerable variation; females produced extra-pair offspring with males to whom they were more or less related than they were to their socially paired male by up to approximately ±0.2, reflecting switches between first-degree and distant inbreeding in both directions (Fig. 1C). Furthermore, *k*_DIFF_ did not differ between females’ extra-pair daughters versus sons (mean effect for sons relative to daughters –0.001 ± 0.001 SE, *t* = –0.8, *P* = 0.44), showing that females did not alter *f* to different degrees in extra-pair daughters versus sons.

### DIFFERENCE IN KINSHIP THROUGH RANDOM EXTRA-PAIR REPRODUCTION

**Figure 2:**
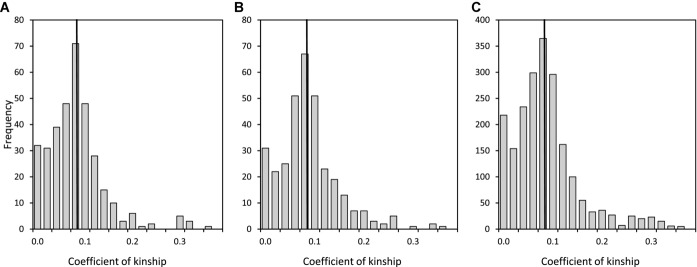
Distributions of the coefficient of kinship (*k*) between a female song sparrow and her (A) first-neighbor, (B) second-neighbor, and (C) non-neighbor males, across 343, 330, and 2080 pairwise comparisons relating to 130 breeding attempts that produced observed extra-pair offspring. Vertical lines demarcate means.

Across the 130 breeding attempts that produced the 216 observed extra-pair offspring, females had means of 4.1 ± 1.6 (range 1–7) first-neighbor males, 3.8 ± 1.5 (range 1–8) second-neighbors, and 25.4 ± 12.3 (range 4–51) non-neighbors. Across all possible female–male pairings defined for these breeding attempts, mean pairwise *k* was 0.089 ± 0.061 (median 0.086) between the female and a first-neighbor male, 0.096 ± 0.062 (median 0.091) with a second-neighbor, and 0.095 ± 0.068 (median 0.087) with a non-neighbor (Fig. 2). Females that produced observed extra-pair offspring were therefore no more or less closely related to first-neighbor males than to second-neighbors or non-neighbors on average (ANOVA on square-root transformed *k*, *F*_2,2750_ = 0.8, *P* = 0.46).

**Figure 3:**
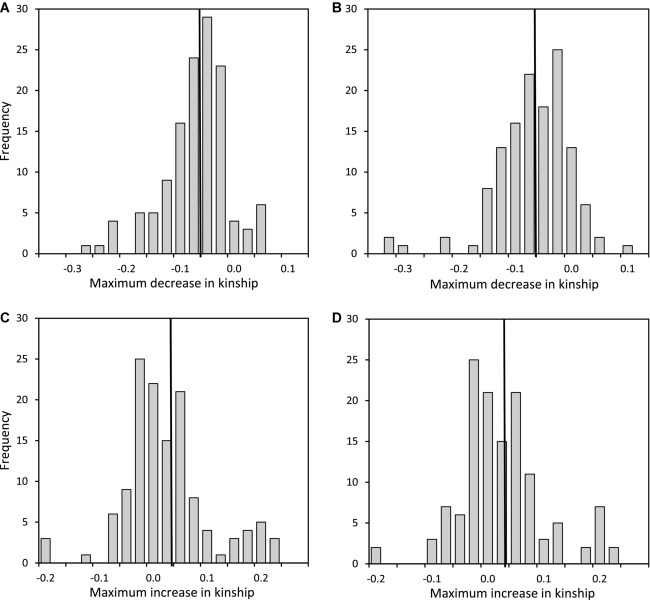
Distributions of the maximum degrees to which females could have reduced their coefficient of kinship (*k*) with the sire of their offspring (and hence reduced offspring *f*) through extra-pair reproduction with a (A) first-neighbor or (B) second-neighbor male (where negative values indicate reduced offspring *f*), or increased *k* (and hence increased offspring *f*) through extra-pair reproduction with a (C) first-neighbor or (D) second-neighbor male (where positive values indicate increased offspring *f*). Vertical lines demarcate means.

The maximum degrees to which females could have reduced *k* with the sire of their offspring (and hence reduced offspring *f*) through extra-pair reproduction with first-neighbor or second-neighbor males averaged 0.058 ± 0.063 (median 0.050) and 0.056 ± 0.066 (median 0.052), respectively (Fig. 3A, B). Meanwhile, the maximum degrees to which females could have increased *k* (and hence increased offspring *f*) through extra-pair reproduction with first-neighbor or second-neighbor males averaged 0.039 ± 0.080 (median 0.024) and 0.038 ± 0.078 (median 0.026), respectively (Fig. 3C, D). Overall, females had opportunity to reduce offspring *f* through extra-pair reproduction with first-neighbor and second-neighbor males in 113 (87%) and 104 (80%) of 130 cases, respectively, and opportunity to increase offspring *f* in 86 (66%) and 87 (67%) cases, respectively. Therefore, most females that produced observed extra-pair offspring had substantial opportunity to increase or decrease offspring *f* through extra-pair reproduction with neighboring males. However, some females had zero opportunity to change offspring *f* in a particular direction (Fig. 3), and on average there was greater opportunity to reduce offspring *f* than to increase it.

**Figure 4:**
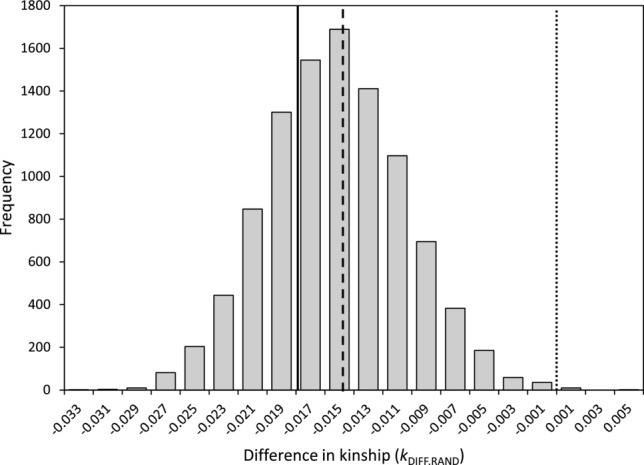
Distribution of the mean difference in coefficient of kinship between a female song sparrow and her socially paired male versus a random extra-pair male (*k*_DIFF.RAND_) across social pairings that produced 216 observed extra-pair offspring, across 10,000 randomizations. Dotted, dashed, and solid lines, respectively demarcate *k*_DIFF.RAND_ = 0, the grand mean *k*_DIFF.RAND_, and the mean observed difference in kinship between a female and her socially paired male versus observed extra-pair male (*k*_DIFF_).

Across all 216 observed extra-pair offspring, the grand mean of the mean randomized *k*_DIFF_ (*k*_DIFF.RAND_) generated by assigning random extra-pair sires to each offspring was –0.015 ± 0.005 (range –0.032 to 0.005, Fig. 4). Overall, 99.9% of mean *k*_DIFF.RAND_ values were negative, and 29.0% were more negative than the observed raw mean *k*_DIFF_ of –0.018 (Fig. 4). The mean *k*_DIFF_ estimated across the 216 observed extra-pair offspring therefore did not differ from that expected given random extra-pair reproduction among females and their neighboring males. Because females were no more or less closely related to first-neighbor males than to second-neighbors or non-neighbors, this conclusion did not change when randomizations were repeated with markedly different probabilities of sampling extra-pair males from different neighbor categories.

### KINSHIP AND THE PROBABILITY OF EXTRA-PAIR REPRODUCTION

There were 301 clutches where ≥1 offspring survived to banding, produced by 90 females and 138 social pairings. Mean *k*_SOC_ was 0.099 ± 0.066 (median 0.091, range 0.000–0.356) across all 301 breeding attempts and 0.100 ± 0.065 (median 0.090, range 0.000–0.356) across the 138 social pairings. Mean clutch size was 3.4 ± 0.7 eggs (median 4, range 1–4), and 83.2% of eggs resulted in banded offspring. Mean brood size was 3.0 ± 1.0 banded offspring (median 3, range 1–4), of which 24.2% were extra-pair offspring.

**Figure 5:**
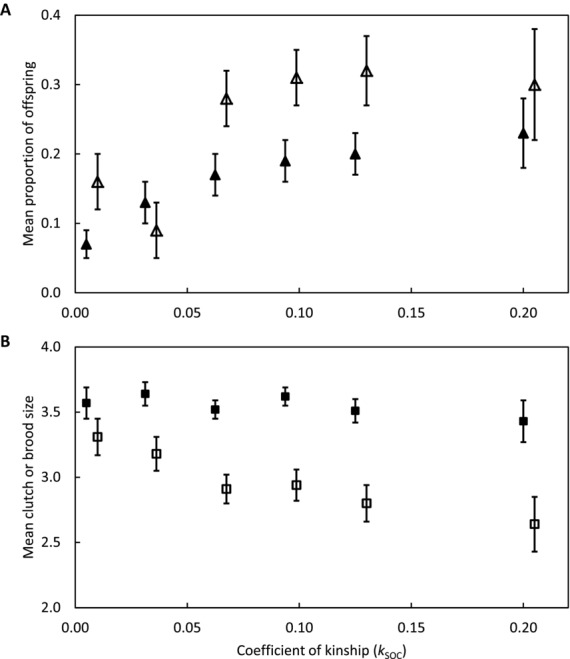
Mean (±1 SE) (A) proportion of banded offspring that were sired by an extra-pair male (open symbols) and proportion of eggs that died before banding (filled symbols), and (B) clutch size (filled symbols) and brood size (open symbols) in relation to a female song sparrow's coefficient of kinship (*k*_SOC_) with her socially paired male. Means were estimated across observed broods pooled into six categories of *k*_SOC_ solely for visualization: *k*_SOC_ < 0.03125 (*N* = 35 broods), 0.03125 ≤ *k*_SOC_ < 0.0625 (*N* = 45), 0.0625 ≤ *k*_SOC_ < 0.09375 (*N* = 75), 0.09375 ≤ *k*_SOC_ < 0.125 (*N* = 69), 0.125 ≤ *k*_SOC_ < 0.20 (*N* = 49), and *k*_SOC_ ≥ 0.20 (*N* = 28).

The probability that a banded offspring would be an extra-pair offspring increased with *k*_SOC_, indicating that the probability of extra-pair reproduction was higher when a female was more closely related to her socially paired male (Table 1A; Fig. 5A). Estimated β_EPR_ was therefore positive and substantial (Table 1A). Clutch size did not vary with *k*_SOC_ (Table 1B; Fig. 5B). However, the probability that an egg would die before banding increased with *k*_SOC_, showing that the probability of failing to observe an offspring's paternity was higher when a female was more closely related to her socially paired male (Table 1C; Fig. 5A). Mean brood size at banding consequently tended to decrease with increasing *k*_SOC_ (Table 1D; Fig. 5B).

**Table 1:** Generalized linear mixed models relating (A) the probability that a banded offspring was sired by an extra-pair male and hence was an extra-pair offspring rather than a within-pair offspring (i.e., the probability of extra-pair reproduction), (B) clutch size, (C) the probability that an assumed conceived offspring (i.e., observed egg) died before banding, and (D) brood size at banding to the coefficient of kinship between a female and her socially paired male (*k*_SOC_) across 301 broods

		Fixed effects				Random effects	
	Intercept ± 1SE	β ± 1SE	*P*	Year	*P*	Female variance	Pair variance
(A) Probability of extra-pair reproduction	−2.22 ± 0.44	β_EPR_ = 7.32 ± 2.68	0.006	−0.37; −0.23; 0.59; 0.69; −0.07	0.36; 0.65; 0.20; 0.18; 0.88	0.22	2.10
(B) Clutch size	1.33 ± 0.09	β_clutch_ = −0.20 ± 0.48	0.67	−0.09; 0.01; −0.03; −0.02; −0.07	0.41; 0.96; 0.77; 0.88; 0.52	<0.01	<0.01
(C) Probability of offspring mortality	−2.75 ± 0.33	β_die_ = 4.24 ± 1.40	0.002	1.10; 0.54; 0.63; 0.81; 0.68	0.002; 0.16; 0.08; 0.04; 0.06	0.20	<0.01
(D) Brood size	1.30 ± 0.09	β_brood_ = −1.01 ± 0.54	0.06	−0.26; −0.06; −0.11;	0.03; 0.61; 0.33;	<0.01	<0.01
				−0.11; −0.15	0.40; 0.17		

β is the slope of each regression on *k*_SOC_ (± 1 SE). Year effects are the contrasts in β from 2007 for 2008–2012, respectively, and associated *P* values.

### BIAS DUE TO EARLY OFFSPRING MORTALITY

In total, 1071 eggs were laid in the 301 focal clutches. Simulations where the paternity of all 1071 assumed conceived offspring was assigned to a female's observed socially paired male or to a random extra-pair male, and where hypothetical failure to observe the assigned paternity was imposed by simulating inbreeding depression in early offspring survival, showed that such observation failure can substantially bias estimates of *k*_DIFF_ and β_EPR_.

**Figure 6:**
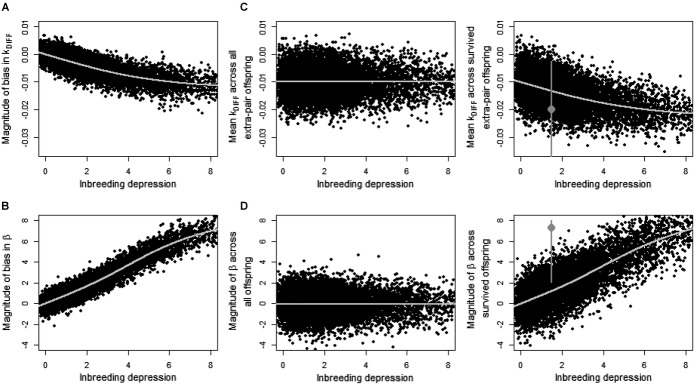
Estimates of the difference in coefficient of kinship (*k*_DIFF_) between a female song sparrow and her socially paired male (*k*_SOC_) versus her extra-pair male (*k*_EP_), the regression of the probability of extra-pair reproduction on *k*_SOC_ (β_EPR_), and the biases in these estimates given increasing inbreeding depression (*B*_ID_) in early survival across simulated offspring. Panels show the magnitude of bias in (A) *k*_DIFF_ (*k*_BIAS_) and (B) β_EPR_ (β_BIAS_) due to observation failure, the true magnitude of (C) *k*_DIFF_ (*k*_DIFF.ALL.SIM_) and (D) β_EPR_ (β_EPR.ALL.SIM_) across all simulated offspring, and the estimated magnitude of (E) *k*_DIFF_ (*k*_DIFF.SURV.SIM_) and (F) β_EPR_ (β_EPR.SURV.SIM_) across offspring that survived to simulated observation. Black points show estimates from each of 10,000 iterations. Lines show fitted general additive models. To facilitate qualitative comparison, gray points show the values of (E) *k*_DIFF_ and (F) β_EPR_ estimated from the real song sparrow data (with 95% confidence intervals), assuming *B*_ID_ = 1.5 haploid lethal equivalents.

Specifically, mean *k*_DIFF_ estimated across simulated offspring that survived to observation (*k*_DIFF.SURV.SIM_) was negatively biased compared to the true value across all simulated extra-pair offspring (*k*_DIFF.ALL.SIM_, Fig. 6A). The absolute magnitude of bias (*k*_BIAS_) was small even given substantial inbreeding depression in early survival (*B*_ID_, Fig. 6A), but large relative to the range of *k* and the potential range of *k*_DIFF_ (Figs. 3). Meanwhile, β_EPR_ estimated across simulated offspring that survived to observation (β_EPR.SURV.SIM_) was positively biased compared to the true value across all simulated offspring (β_EPR.ALL.SIM_), and the magnitude of bias (β_BIAS_) was substantial given moderate or high *B*_ID_ (Fig. 6B).

The simulations also showed that when extra-pair paternity was randomly assigned, mean *k*_DIFF.ALL.SIM_ measured across all conceived extra-pair offspring was typically negative (–0.010 ± 0.005, 97.7% of values were negative) and independent of *B*_ID_ (Fig. 6C). Mean β_EPR.ALL.SURV_ measured across all conceived offspring was close to zero (–0.03 ± 1.12, 51% of values were negative) and independent of *B*_ID_, but showed substantial sampling variance (Fig. 6D). These values of *k*_DIFF.ALL.SIM_ and β_EPR.ALL.SURV_ contain no bias due to observation failure. The negative mean *k*_DIFF.ALL.SIM_ (Fig. 6C) therefore shows that female song sparrows would on average reduce their *k* with the sire of their offspring (and hence reduce offspring *f*) through purely random extra-pair reproduction given the distributions of *k*_SOC_ and *k*_EP_ between females and their observed socially paired males versus their potential extra-pair males.

Due to the combination of intrinsic structure and sampling variance (Fig. 6C, D) and bias stemming from observation failure (Fig. 6A, B), mean *k*_DIFF.SURV.SIM_ and β_EPR.SURV.SIM_ estimated across simulated offspring that survived to hypothetical observation were typically negative and positive, respectively (Fig. 6E, F). Mean estimated *k*_DIFF.SURV.SIM_ was commonly about –0.01to –0.02 (Fig. 6E), whereas β_EPR.SURV.SIM_ could be substantial given moderate or high *B*_ID_ (Fig. 6F).

## Discussion

Extra-pair reproduction is widely hypothesized to allow socially paired females to reduce their relatedness to the sire of their offspring, thereby reducing offspring inbreeding level and consequent inbreeding depression in offspring fitness (Jennions and Petrie 2000; Tregenza and Wedell 2000; Kempenaers 2007). However, field studies aiming to relate extra-pair reproduction to inbreeding and relatedness rarely use sufficient pedigree or genotypic data to measure subtle variation in expected kinship (*k*) or realized relatedness between a female and her actual and potential mates, or to measure offspring coefficient of inbreeding (*f*) or genome-wide heterozygosity, with high accuracy or precision. Furthermore, such studies do not generally consider biases stemming from failure to observe inbred offspring that die before genotyping and paternity assignment and hence before *f*, *k*, heterozygosity, or relatedness can be estimated.

The comprehensive pedigree available for Mandarte's song sparrows allowed unusually precise estimation of *k* among interacting females and males and *f* of resulting offspring, and hence of expected identity by descent (Supporting Information). Pedigree analyses demonstrated substantial variation in *k* among actual and potential mates, and hence substantial opportunity for females to decrease or increase offspring *f* through extra-pair reproduction (Figs. 1, 3). In practice, across all observed extra-pair offspring (i.e., that survived to posthatch DNA sampling and paternity assignment), females on average slightly reduced their *k* with the offspring's sire, and hence slightly reduced the *f* of extra-pair offspring compared to their alternative within-pair offspring. The mean reduction was small in absolute terms (*k*_DIFF_ ≈ –0.02), but constitutes an 18% reduction relative to the mean *f* of 0.11 of females’ within-pair offspring. This reduction could nontrivially increase offspring fitness given strong inbreeding depression (as is widely estimated, including in the focal song sparrow population; Charlesworth and Charlesworth 1999; Keller and Waller 2002; Reid et al. 2014).

However, there was substantial variation in *k*_DIFF_ across observed extra-pair offspring and no universal directional change; some extra-pair offspring had substantially higher and lower *f* values than the female's alternative within-pair offspring (Fig. 1C). Furthermore, female song sparrows did not reduce the *f* of observed extra-pair offspring any more (or less) than expected given random extra-pair reproduction with neighboring males, or with the wider male population. Therefore, across females that produced observed extra-pair offspring, there was no evidence of inbreeding avoidance or preference defined as deviations from random extra-pair reproduction.

The females that produced observed extra-pair offspring were not significantly less (or more) closely related to first-neighbor males than to less proximate potential extra-pair males, suggesting that the slight reduction in mean *k* and hence offspring *f* that simulations predicted would result from random extra-pair reproduction among neighbors did not simply reflect small-scale spatial variation in *k* (e.g., Foerster et al. 2003; Brouwer et al. 2011). Rather, because the probability of extra-pair reproduction increased with a female's *k* with her socially paired male, females that produced observed extra-pair offspring were relatively closely related to their socially paired males (Table 1A; Fig. 5A). Randomly chosen extra-pair males were therefore less closely related to that female on average. Similarly higher probabilities of extra-pair reproduction by females that are more closely related to their socially paired males have been reported in other systems, and interpreted as evidence of adaptive inbreeding avoidance through some form of pre- or postcopulatory sexual selection (e.g., Blomqvist et al. 2002; Suter et al. 2007; Cohas et al. 2008; Brouwer et al. 2011; Varian-Ramos and Webster 2012; Kingma et al. 2013).

### BIAS DUE TO EARLY OFFSPRING MORTALITY

However, rather than indicating strategic inbreeding avoidance, spurious evidence of a reduction in mean offspring *f* through extra-pair reproduction (i.e., *k*_DIFF_ < 0) and increasing probability of extra-pair reproduction with increasing *k*_SOC_ (i.e., β_EPR_ > 0) could potentially result from failure to observe the paternity of inbred offspring that die during early development. Indeed, the probability that a song sparrow egg would die before DNA sampling and paternity assignment at six days posthatch was higher in clutches produced by more closely related socially paired parents (Table 1C; Fig. 5A), and inbreeding depression in hatching success and early survival is widely observed (Lynch and Walsh 1998; Hemmings et al. 2012). Simulations that randomly assigned within-pair or extra-pair sires to all eggs laid in focal song sparrow nests, and then imposed inbreeding depression in offspring survival to hypothetical observation of the assigned paternity, readily generated negative bias in *k*_DIFF_ and substantial positive bias in β_EPR_ estimated across offspring that were simulated to survive to observation (Fig. 6A, B).

The cause of such bias is intuitive given the restricted range of *k* arising in populations with biparental sexual reproduction and no obligate close inbreeding. Given random extra-pair reproduction with respect to both *k*_SOC_ and *k*_EP_ and inbreeding depression in early survival, extra-pair offspring produced by females with high *k*_SOC_ will on average have lower *f* than the female's inbred within-pair offspring and consequently be more likely to survive to observation. Conversely, extra-pair offspring produced by females with low *k*_SOC_ will on average have higher *f* than the female's outbred within-pair offspring and hence be less likely to survive. The probability that a female's offspring will be sired by an extra-pair male will therefore be overestimated to a degree that increases with increasing *k*_SOC_. Furthermore, extra-pair offspring with negative *k*_DIFF_ are likely to be less inbred on average than extra-pair offspring with positive *k*_DIFF_ and therefore be more likely to survive to observation, causing mean estimated *k*_DIFF_ to be negatively biased.

Such biases are hard to eliminate if the paternity or heterozygosity of offspring that die during early development cannot be observed. However, when the number of unobserved offspring can be estimated, for example by comparing clutch and brood sizes, one approach is to simulate the potential biases in *k*_DIFF_ and β_EPR_ that could result from observation failure given postulated magnitudes of inbreeding depression in early survival. Empirical estimates of *k*_DIFF_ and β_EPR_ can then be compared to simulated estimates and bias, allowing some consideration of whether true underlying effects might differ from zero.

Any such comparison requires the magnitude of inbreeding depression in offspring survival to observation of paternity (*B*_ID_) to be estimated. This creates a further empirical difficulty, because *B*_ID_ cannot be directly estimated when *f* is unknown for offspring that die before paternity can be observed. However, data from a sample of genotyped song sparrow offspring that died before standard paternity assignment, and further simulations that quantified bias in *B*_ID_ estimated from observed social paternity rather than unobserved genetic paternity, suggest that inbreeding depression in survival from conception to banding is roughly 1.5 haploid lethal equivalents in the focal song sparrow population (Supporting Information). The value of mean *k*_DIFF_ ≈ –0.02 estimated across observed song sparrow offspring then falls within the range that basic simulations predict could be readily generated by random extra-pair reproduction plus bias stemming from failure to observe inbred extra-pair offspring that died early (Fig. 6E). Therefore, after considering bias stemming from inbreeding depression in early offspring survival, there is no compelling evidence that polyandrous female song sparrows actively adjust offspring *f* through extra-pair reproduction through any nonrandom “inbreeding strategy.”

In contrast, the value of β_EPR_ = 7.3 estimated across observed song sparrow offspring falls outside the range that basic simulations predict could result from sampling variance and bias due to observation failure given purely random extra-pair reproduction and inbreeding depression of *B*_ID_ ≈ 1.5 (Fig. 6F). However, the 95% confidence interval for the empirical estimate of β_EPR_ includes simulated values that arose given random extra-pair reproduction, and the estimated β_EPR_ fell within the range of bias that could arise if *B*_ID_ was in fact higher than estimated (Fig. 6F). The evidence that female song sparrows that are socially paired to more closely related males are more likely to produce extra-pair offspring is therefore best viewed as equivocal; the null hypothesis that extra-pair reproduction is random with respect to *k*_SOC_ cannot be definitively rejected. Firmer conclusions would require simulations that quantitatively capture all major processes underlying variation in female extra-pair reproduction and accurate estimation of *B*_ID_, requiring greater knowledge than is currently available for any wild population.

Further simulations are required to quantify the degree to which early offspring mortality and consequent failure to observe paternity might bias estimates of *k*_DIFF_ and β_EPR_ in other systems with different life histories, but the problem seems likely to be general. Bias could be minimized by increasing efforts to sample and assign paternity to all conceived offspring (e.g., Olsson et al. 1999; García-González 2008), but is unlikely to be eliminated simply by data censoring such as restricting statistical analyses to broods where the paternity of all offspring was observed (e.g., Tarvin et al. 2005; Brouwer et al. 2011; Kingma et al. 2013). This is because, given inbreeding depression in early survival, the probability of completely observing paternity will depend on *k*_SOC_ and *k*_EP_ (Supporting Information). Previous and future studies that report that females reduce offspring *f* (or homozygosity) through extra-pair reproduction, or that extra-pair reproduction is more frequent when females are more closely related to their socially paired male, might therefore need to be (re)evaluated in the light of bias stemming from preobservation offspring mortality. The degree to which previously published estimates might be biased is hard to assess because the number and distribution of eggs or offspring for whom inbreeding, heterozygosity, or parental relatedness was not estimated is rarely reported; future studies could usefully provide such information (see also Olsson et al. 1999).

### FITNESS CONSEQUENCES OF EXTRA-PAIR REPRODUCTION

Simulations showed that, in song sparrows, the true mean *k*_DIFF_ (i.e., with zero bias due to failure to observe paternity) was almost always negative given random extra-pair reproduction (mean *k*_DIFF_ ≈ –0.01, Fig. 6C). This implies that, in the focal song sparrow population, random extra-pair reproduction would on average reduce a female's *k* with the sire of her offspring and hence reduce mean offspring *f*. This reduction arose because females were on average slightly more closely related to their socially paired male than to their potential extra-pair males. Given substantial inbreeding depression in fitness, as estimated in song sparrows (Reid et al. 2014), the slight reduction in mean *f* of extra-pair offspring resulting from random extra-pair reproduction would itself increase female fitness defined as the number of allele copies expected to be present identical by descent in grand-offspring. Therefore, female song sparrows might on average slightly increase their fitness through random rather than any form of actively strategic extra-pair reproduction.

In contrast, extra-pair song sparrow offspring hatched during 1993–2003 tended to have lower lifetime reproductive success than their within-pair maternal half-siblings (Sardell et al. 2012). If mean *k*_DIFF_ for these offspring were negative (as observed for offspring hatched during 2007–2012, meaning that extra-pair offspring averaged slightly less inbred than their within-pair maternal half-siblings), then some other genetic or environmental component of fitness must differ between maternal half-sibs and cause the lower lifetime reproductive success of extra-pair offspring. Indeed, extra-pair sires had lower additive genetic values for juvenile survival than within-pair sires on average (Reid and Sardell 2012). Different genetic components of fitness are therefore differentially influenced by extra-pair reproduction.

## Conclusion

Despite the widespread presumption that inbreeding should be avoided (Tregenza and Wedell 2000; Jamieson et al. 2009), there is little explicit theory predicting whether active inbreeding avoidance or preference is likely to evolve in species whose ecologies and reproductive systems mean that biparental inbreeding among diverse relatives is potentially common (even if precise kin recognition were feasible), or hence whether inbreeding avoidance could drive evolution of extra-pair reproduction or polyandry. In song sparrows, where inbreeding depression in fitness is substantial (Reid et al. 2014), females had considerable opportunity to reduce or increase offspring *f* through strategic extra-pair reproduction with neighboring males. However, the most parsimonious interpretation of the data is that females most probably do not exhibit nonrandom inbreeding avoidance or preference through extra-pair reproduction, but that females still slightly reduce mean offspring *f* through random extra-pair reproduction.

Such reductions in offspring *f* could stem from nonrandom formation or persistence of social pairings in relation to *k*_SOC_ (Reid et al. 2015). However, even if such reductions were to occur more generally, they seem likely to be small and hard to detect. Across observed song sparrow extra-pair offspring, the magnitude of error in estimated *k*_DIFF_ due to incorrectly assigned ancestors was smaller than the best estimate of mean *k*_DIFF_, and than the simulated true *k*_DIFF_ stemming from random extra-pair reproduction, only when analyses were restricted to offspring whose potential great-great-grandparents were all accurately known (Supporting Information). This degree of precision is currently beyond most pedigree-based (and marker-based) estimates of *k* or relatedness in wild populations.

Furthermore, even with 20 years of complete genetic pedigree data, the relationship between extra-pair reproduction and *k*_SOC_ (β_EPR_) was estimated with substantial uncertainty (Table 1A). Simulations showed that, due to the combination of sampling variance and bias, a large range of β_EPR_ values could be estimated given a true value of zero (Fig. 6F). The diverse effects reported by existing field studies (Kempenaers 2007; Jamieson et al. 2009; Szulkin et al. 2013) might therefore stem partly from sampling variance, and from (co)variation in life history and inbreeding depression in early offspring survival and consequent failure to observe paternity, rather than from variation in inbreeding avoidance or preference. Our results imply that empiricists will need to invest even more heavily in collecting high-quality relatedness data to adequately quantify subtle variation in inbreeding strategy, and hence test key hypotheses explaining extra-pair reproduction.

## DATA ARCHIVING

The doi for our data is 10.5061/dryad.f9013.

## Supporting Information

**Appendix 1**. Magnitude of error in the estimated difference between a female's coefficient of kinship with her socially–paired male versus extra–pair male(s) with increasing generations of pedigree correction. **Appendix 2**. Estimating inbreeding depression in early survival. **Appendix 3**. Bias in estimates of *k*_DIFF_ and β_EPR_ when analyses are restricted to breeding attempts where the paternity of all offspring is observed.
