# Supplementary material for: Quantifying inbreeding avoidance through extra-pair reproduction
Source: Evolution. 2014 Dec 3;69(1):59–74. doi: 10.1111/evo.12557 (PMC4312944; doi:10.1111/evo.12557)
Supplement: Supplementary file 1 [file evo0069-0059-sd1.docx]

**Supporting Information**

**QUANTIFYING INBREEDING AVOIDANCE THROUGH EXTRA-PAIR REPRODUCTION**

**Jane M. Reid, Peter Arcese, Lukas F. Keller, Ryan R. Germain, A. Bradley Duthie, Sylvain Losdat, Matthew E. Wolak and Pirmin Nietlisbach**

***Magnitude of error in the estimated difference between a female’s coefficient of kinship with her socially-paired male versus extra-pair male(s) with increasing generations of pedigree correction***

One objective was to estimate the difference between a female song sparrow’s coefficient of kinship with her socially-paired male versus her observed extra-pair male(s) (*k*_DIFF_ = *k*_EP_ – *k*_SOC_) from pedigree data. Pedigree data spanning 1975 to 2012 were initially compiled based on observed parental behaviour. Paternity of individuals hatched during 1993-2012 was then genetically verified, and the corresponding pedigree corrected for extra-pair paternity (Reid et al. 2014). However, the pedigree for 1975-1992 presumably still contains some paternity error, introducing error into estimates of *k*_SOC_, *k*_EP_ and *k*_DIFF_ between sparrows breeding subsequently.

To minimise such error, analyses were restricted to trios of females and their socially-paired and extra-pair males whose great-grandparents (i.e. the great-great-grandparents of resulting offspring) were all genetically verified or immigrants (or the ancestors of immigrants, Fig. S1). However, some great-great-grandparents and more distant ancestors of the focal breeding adults (i.e. great-great-great-grandparents of focal offspring) are presumably still mis-assigned in the complete pedigree due to unobserved extra-pair paternity prior to 1993. Error stemming from mis-assigned great-great-great-grandparents should be small because the impact of any mis-assigned ancestor on *k*_SOC_ and *k*_EP_ among contemporary individuals is expected to decrease non-linearly with increasing intervening generations (Balloux et al. 2004; Slate et al. 2004). However the exact impact of any individual mis-assigned ancestor cannot be readily predicted in populations with irregular inbreeding, and will depend on the degree to which mis-assignment alters intervening pedigree loops. Moreover, error in estimates of *k*_DIFF_ will be greater than the errors in *k*_SOC_ and *k*_EP_ when the absolute difference between the latter two errors is big. Remaining error in *k*_DIFF_ could then be non-negligible compared to the average degree to which females alter the coefficient of inbreeding (*f*) of offspring through extra-pair reproduction, and hence compared to the true value of *k*_DIFF_.

To assess the likely magnitude of error in estimates of *k*_SOC_, *k*_EP_ and *k*_DIFF_ due to remaining historical pedigree error, we quantified the errors given increasing generations of pedigree correction. We calculated *k*_DIFF_ across all extra-pair offspring hatched in 2011 and 2012 based on the full social pedigree, and then based on pedigrees that were partially corrected for extra-pair paternity such that females that produced offspring in 2011 or 2012 and their socially-paired and extra-pair males had genetically corrected ancestors up to and including their i) parents, ii) grandparents, iii) great-grandparents and iv) great-great-grandparents (meaning that the focal extra-pair offspring had verified ancestors up to and including one further generation back at each level, Fig. S1). Finally, we estimated *k*_DIFF_ across these same extra-pair offspring from the full corrected pedigree. We then calculated the absolute magnitude of the difference in *k*_DIFF_ estimated from the full corrected pedigree versus the social pedigree and the set of partially corrected pedigrees, thereby quantifying the degree to which each additional generation of pedigree correction reduced error in estimated *k*_DIFF_.

Mean *k*_DIFF_ calculated from the full corrected pedigree for 74 extra-pair offspring hatched in 2011 or 2012 was -0.0251 ± 0.0766SD (median -0.0029, range -0.2409-0.0826). The mean absolute magnitude of *k*_DIFF_ across these same 74 extra-pair offspring was 0.0559 (median 0.0311, range 0.0000-0.2410). The mean and maximum magnitudes of error that paternity error introduced into estimates of *k*_DIFF_ were substantial if *k*_DIFF_ was estimated from pedigree data that were uncorrected for paternity error in any assigned ancestors of the focal breeding females and males (i.e. the parents of the observed extra-pair offspring, Table S1A). However these magnitudes decreased rapidly with increasing generations of paternity correction, and tended towards zero when all ancestors up to and including the great-great-grandparents of focal adults were correctly assigned (Table S1B-E). The proportional magnitude of error in *k*_DIFF_ due to paternity error therefore decreased from 84% with no paternity correction to <5% when the paternity of all ancestors up to the great-grandparents of focal adults was verified. These differences are calculated relative to the full song sparrow pedigree for 1975-2012, which still contains paternity error during 1975-1992. However these analyses demonstrate that error in *k*_DIFF_ becomes small given 3-4 generations of pedigree correction. With correction to great-grandparents, remaining error is an order of magnitude smaller than the estimated effect.

**Figure S1.** Analyses were restricted to cases where all 24 great-grandparents of the focal female and her socially-paired and extra-pair male (i.e. the great-great-grandparents of resulting offspring) were known and genetically verified, or were immigrants to the focal population (or the ancestors of these immigrants) and hence defined as unrelated. Some ancestors of the focal parents will be identical when there is inbreeding.


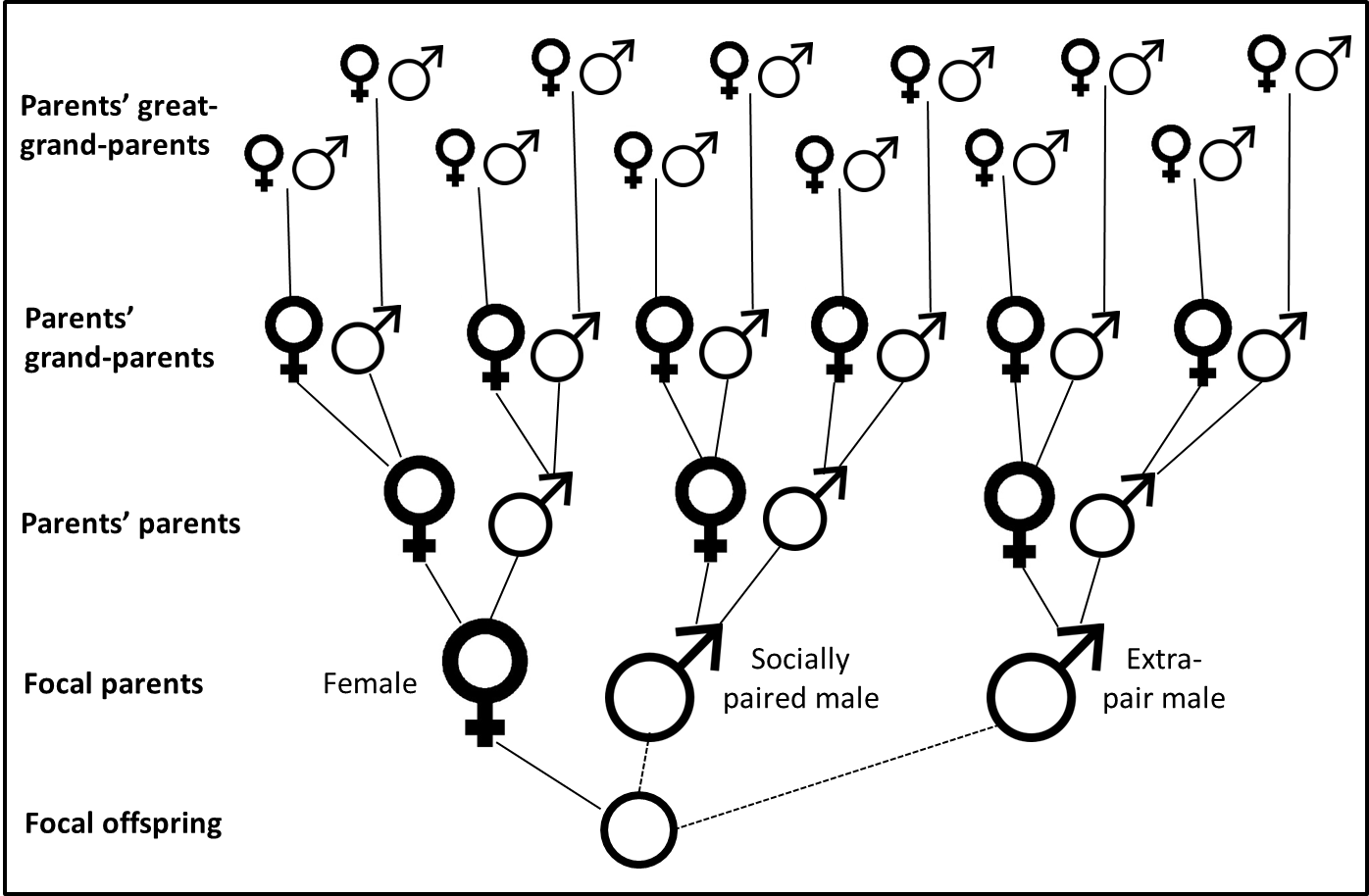


**Table S1.** Statistics summarising the absolute magnitude of the difference in coefficient of kinship (*k*_DIFF_) between a female and her socially-paired versus extra-pair male(s) across all extra-pair offspring hatched in 2011 and 2012, and the magnitude of the difference in *k*_SOC_ and *k*_EP_ between a female and these males respectively, calculated from the full corrected pedigree versus the A) full social pedigree, or partially corrected pedigrees where all adults breeding in 2011 or 2012 were assigned their true genetic ancestors up to and including their B) parents, C) grandparents, D) great-grandparents and E) great-great-grandparents. The percentages of the mean magnitudes of *k*_DIFF_, *k*_SOC_ and *k*_EP_ of 0.0559, 0.1175 and 0.0924 estimated from the full corrected pedigree that was represented by the difference in *k*_DIFF_ at each level of pedigree correction are also shown. IQR is the inter-quartile range.

|  | *k*_DIFF_ | | *k*_SOC_ | | *k*_EP_ | |
| --- | --- | --- | --- | --- | --- | --- |
|  | Mean  (Median) | IQR  (Range) | Mean  (Median) | IQR  (Range) | Mean  (Median) | IQR  (Range) |
| A) Full social pedigree | 0.0472  (0.0349)  84.4% | 0.0138 – 0.0571  (0.0000 – 0.1674) | 0.0311  (0.0289)  26.4% | 0.0074 – 0.0433  (0.0000 – 0.0923) | 0.0292  (0.0167)  31.6% | 0.0064 – 0.0356  (0.0000 – 0.1738) |
| B) Parents | 0.0149  (0.0107)  26.7% | 0.0040 – 0.0190  (0.0000 – 0.0743) | 0.0121  (0.0076)  10.3% | 0.0026 – 0.0134  (0.0000 – 0.0698) | 0.0082  (0.0070)  8.9% | 0.0042 – 0.0096  (0.0000 – 0.0276) |
| C) Grandparents | 0.0104  (0.0040)  18.6% | 0.0018 – 0.0127  (0.0000 – 0.0521) | 0.0086  (0.0055)  7.3% | 0.0021 – 0.0075  (0.0000 – 0.0411) | 0.0058  (0.0055)  6.3% | 0.0027 – 0.0075  (0.0000 – 0.0269) |
| D) Great-grandparents | 0.0024  (0.0012)  4.3% | 0.0002 – 0.0035  (0.0000 – 0.0168) | 0.0020  (0.0018)  1.7% | 0.0006 – 0.0032  (0.0000 – 0.0061) | 0.0024  (0.0018)  2.6% | 0.0006 – 0.0034  (0.0000 – 0.0126) |
| E) Great-great-grandparents | 0.0004  (0.0003)  0.7% | 0.0001 – 0.0004  (0.0000 – 0.0015) | 0.0005  (0.0004)  0.4% | 0.0002 – 0.0006  (0.0000 – 0.0024) | 0.0007  (0.0005)  0.8% | 0.0003 – 0.0009  (0.0000 – 0.0033) |

***Estimating inbreeding depression in early survival***

In general, inbreeding depression in early embryonic survival might be expected to be strong, reflecting lethal effects of deleterious recessive alleles that affect key developmental processes (Olsson et al. 1999; Keller and Waller 2002). Such strong inbreeding depression has been observed in diverse systems (Lynch and Walsh 1998; Hemmings et al. 2012). However the magnitude of inbreeding depression in early survival is hard to estimate in wild populations where individuals’ paternity, and hence their coefficient of inbreeding (*f*), cannot be determined until DNA can be sampled at some point post-hatch or post-birth. Inbreeding depression in survival to sampling cannot then be directly estimated because *f* is unknown for individuals that die before sampling.

One partial solution is to determine the paternity, and hence *f* values, of individuals that die before standard sampling but for whom DNA samples can still be collected. In the focal song sparrow population, Taylor et al. (2010) genotyped and assigned paternity to 51 individuals that died before standard DNA sampling at ca. six days post-hatch during 1994-2008. These dead individuals averaged slightly more inbred than their surviving brood-mates, providing evidence of inbreeding depression in early survival (Taylor et al. 2010). During 2009-2013 a further 24 individuals that died before standard sampling were collected and genotyped. Analyses of 65 dead individuals and their surviving brood-mates that had adequate depth of verified pedigree provided an estimate of inbreeding depression in early survival of *B*_sample_ ≈ 1.3 ± 1.1SE haploid lethal equivalents (and hence ca. 2.6 diploid lethal equivalents per zygote). Here, *B*_sample_ was estimated as the slope of a regression of ln(S_S_) on *f*_o_ (i.e. ln(S_S_) = A - *B*_sample_.*f*_o_, Morton et al. 1956; Lynch and Walsh 1998 p.278), where S_S_ is the proportion of all DNA-sampled offspring that survived to banding within each of ten categories of *f* (defined to contain approximately equal numbers of offspring) and *f*_o_ is mean *f* within each category.

However these analyses only included individuals that survived to be late-stage embryos or young hatched chicks, allowing DNA to be extracted from field-collected specimens. They therefore do not capture inbreeding depression in very early embryonic survival (i.e. through the first days of development). A complementary approach is therefore to estimate inbreeding depression in survival to banding across all eggs laid in clutches during 2007-2012. This approach has the advantage of including all conceived individuals (assuming that all eggs are fertile), and of ensuring adequate pedigree depth. However it has the major disadvantage that, due to the inability to observe the paternity of individuals that died before sampling, *f* of all conceived individuals (i.e. eggs) has to be assumed to equal the coefficient of kinship between their observed socially-paired parents (*k*_SOC_). This assumption will be violated when there is extra-pair reproduction.

Nevertheless, across the 301 song sparrow clutches where ≥1 offspring survived to banding, the slope of the regression of the (logit) probability that an egg would survive to banding on *k*_SOC_ was -4.2 ± 1.4SE (Table 1C). The equivalent slope estimated across all 365 clutches (produced by 90 different females and 139 different social pairings) observed during 2007-2012 including those where all offspring died before banding was -3.8 ± 1.6SE. This latter figure translates into estimated inbreeding depression in survival from conception to banding (*B*_kSOC_) of ca. 1.1 ± 0.3 haploid lethal equivalents (and hence ca. 2.2 diploid lethal equivalents). Here, *B*_kSOC_ was estimated as the slope of a regression of ln(S_o_) on *k*_SOCo_ (i.e. ln(S_o_) = A - *B*_kSOC_.*k*_SOCo_, Morton et al. 1956; Lynch and Walsh 1998 p.278), where S_o_ is the observed proportion of eggs from which offspring survived to banding within each of ten categories defined with respect to the kinship between the egg’s socially-paired parents (*k*_SOC_), and *k*_SOCo_ is mean *k*_SOC_ across all eggs within each category (which equals mean *f* of these eggs as estimated from their observed social parentage). The ten categories of *k*_SOC_ were defined to contain approximately equal numbers of eggs. The estimate of inbreeding depression remained similar when estimated with iteratively weighted regression (as recommended by Morton et al. 1956, *B*_kSOC_ = 1.2), and when the analysis was repeated with 20 categories of *k*_SOC_ rather than 10 (*B*_kSOC_ = 1.1).

We then used simulations to quantify the magnitude of bias in this estimate of inbreeding depression in early survival that could be caused by error in estimated *f* for individuals that died before DNA sampling (and hence whose true paternity was unknown). Within the simulations described in the main paper, the magnitude of inbreeding depression in early survival of each conceived individual was calculated by regressing (log) simulated survival on the *f* of individual offspring, thereby quantifying inbreeding depression in early survival (*B*_ID_) across all conceived individuals without error. Within each iteration of the same simulation, we additionally regressed simulated survival of all individuals (i.e. eggs) on *k*_SOC_ between the egg’s mother and her observed socially-paired male, thereby estimating the magnitude of inbreeding depression in early survival in relation to parental *k*_SOC_ (*B*_kSOC_) rather than in relation to true *f* of individual offspring (*B*_ID_). The difference between these two estimates of inbreeding depression in early survival represents the bias caused by failure to observe the true paternity of individuals that died early.

These simulations showed that failure to observe paternity (and hence *f*) of offspring that died early caused inbreeding depression in early survival estimated from parent-level data (*B*_kSOC_) to be downwardly biased compared to the true value calculated across complete individual-level data (*B*_ID_, Fig. S2). Specifically, *B*_kSOC_ commonly underestimated *B*_ID_ by up to 50% (Fig. S2).

These simulations make multiple simplifying assumptions, including that extra-pair reproduction is random with respect to kinship, meaning that strict quantitative conclusions should not be drawn. However the simulations indicate that the magnitude of inbreeding depression in survival from conception to banding might commonly be up to ca. 1.5 times that estimated from parent-level analyses (Fig. S2). The value of *B*_kSOC_ ≈ 1.1 haploid lethal equivalents estimated in song sparrows might therefore equate to true inbreeding depression of the order of *B*_ID_ ≈ 1.1 - 1.5 haploid lethal equivalents (Fig. S2), closely matching the value of *B*_sample_ ≈ 1.3 estimated across dead offspring from which DNA was collected. However, the standard errors associated with these estimates mean that true *B*_ID_ could be as high as 2-3 lethal equivalents. This broad range of values is similar to that estimated in other systems (e.g. Lynch and Walsh 1998, p.279).

**Figure S2.** Relationship between the magnitude of inbreeding depression in simulated offspring survival from conception to banding estimated in relation to kinship between a female and her socially-paired male (*B_k_*_SOC_) versus the true magnitude of inbreeding depression (*B*_ID_) estimated in relation to each individual offspring’s coefficient of inbreeding. Black points relate the estimated inbreeding depression (*B_k_*_SOC_) to the true inbreeding depression (*B*_ID_) in each of 10000 simulations. Solid and dashed lines respectively show the 1:1 and 1:1.5 lines of proportion for *B_k_*_SOC_.


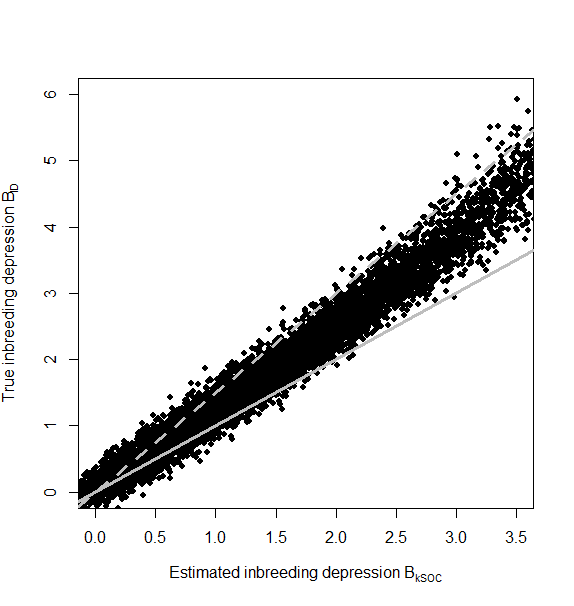


***Bias in estimates of k_DIFF_ and* β*_EPR_ when analyses are restricted to breeding attempts where the paternity of all offspring is observed***

Simulations applied to the song sparrow dataset showed that estimates of the mean difference in coefficient of kinship between a female and her socially-paired versus extra-pair male (*k*_DIFF_ = *k*_EP_ - *k*_SOC_), and of the regression of the (logit) probability of extra-pair reproduction on *k*_SOC_ (β_EPR_), can be substantially biased by failure to observe relatively inbred offspring that die before paternity can be assigned (and hence before *k*_EP_ and *k*_SOC_ can be estimated). One potential response to this problem might be to restrict analyses to breeding attempts where all conceived offspring survived to paternity assignment (i.e. where observed brood size equals the original full clutch size, e.g. Tarvin et al. 2005; Brouwer et al. 2011; Kingma et al. 2013). However, given inbreeding depression in early offspring survival, the probability of observing a complete brood will depend on *k*_SOC_ and *k*_EP_. The broods that are retained in analyses are then likely to be biased towards females that do not produce inbred extra-pair offspring or within-pair offspring, and hence that have low *k*_SOC_ and *k*_EP_ and consequently small *k*_DIFF_. Estimates of β_EPR_, mean *k*_DIFF_ and the variances in *k*_SOC_, *k*_EP_ and/or *k*_DIFF_ might then still be biased, potentially biasing tests of the hypotheses that β_EPR_ and mean *k*_DIFF_ differ from zero.

Indeed, further simulations based on the song sparrow dataset showed that when analyses were restricted to broods where all offspring survived to simulated observation of paternity, β_EPR_ was positively biased compared to true β_EPR_ estimated across all simulated offspring to a degree that increased with the magnitude of inbreeding depression in early offspring survival (*B*_ID_, Fig. S3A). Mean *k*_DIFF_ estimated across extra-pair offspring in completely observed broods was slightly positively biased compared to true mean *k*_DIFF_, at least given moderate *B*_ID_ (Fig. S3B). However estimates of the variance in *k*_DIFF_ were downwardly biased (Fig. S3C). Tests of the hypothesis that mean *k*_DIFF_ < 0 based on analyses of broods where all offspring survived to observation might therefore be anti-conservative.

These simulations suggest that, at least given the song sparrow data structure, unbiased estimates and hypothesis tests regarding *k*_DIFF_ and β_EPR_ cannot be obtained simply by restricting statistical analyses to completely observed broods. Further simulations are required to determine the degree to which such biases might arise more generally. However such biases seem likely to be larger in species with larger clutch sizes and/or lower mean survival to sampling, meaning that the probability of observing the paternity of complete broods will be low.

**Figure S3.** Relationships between the magnitude of inbreeding depression (*B*_ID_) in early offspring survival and bias in A) the estimated regression of the (logit) probability of extra-pair reproduction on a female’s coefficient of kinship (*k*_SOC_) with her socially-paired male (β_EPR_), B) the mean difference (*k*_DIFF_) in kinship between a female and her socially-paired versus extra-pair male (*k*_DIFF_ = *k*_EP_ - *k*_SOC_), and C) the variance in *k*_DIFF_ across all extra-pair offspring when analyses were restricted to broods where all offspring survived to simulated observation of paternity. Black points show values from each of 10000 simulations. Lines show fitted general additive models.


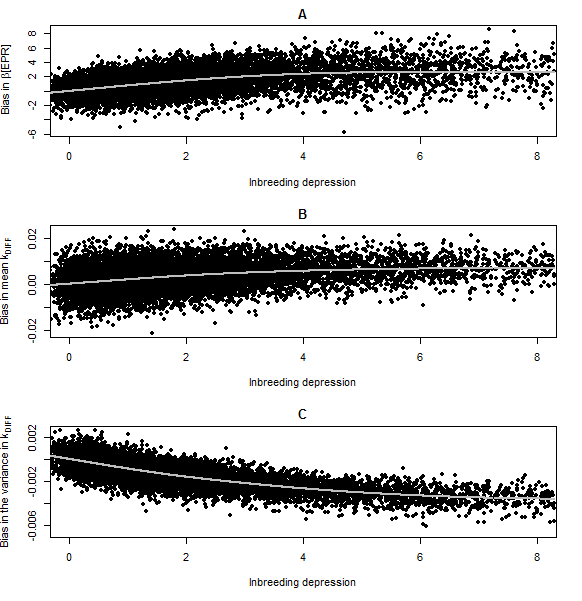


**LITERATURE CITED**

Balloux, F., W. Amos, and T. Coulson. 2004. Does heterozygosity estimate inbreeding in real populations? Mol. Ecol. 13:3021-3031.

Brouwer, L., M. van de Pol, E. Atema, and A. Cockburn. 2011. Strategic promiscuity helps avoid inbreeding at multiple levels in a cooperative breeder where both sexes are philopatric. Mol. Ecol. 20:4796-4807.

Kingma, S. A., M. L. Hall, and A. Peters. 2013. Breeding synchronisation facilitates extrapair mating for inbreeding avoidance. Behav. Ecol. 24:1390-1397.

Lynch, M., and B. Walsh. 1998. Genetics and analysis of quantitative traits. Sinauer, Sunderland.

Reid, J. M., L. F. Keller, A. B. Marr, P. Nietlisbach, R. J. Sardell, and P. Arcese. 2014. Pedigree error due to extra-pair reproduction substantially biases estimates of inbreeding depression. Evolution 68:802-815.

Slate, J., P. David, K. G. Dodds, B. A. Veenvliet, B. C. Glass, T. E. Broad, and J. C. McEwan. 2004. Understanding the relationship between the inbreeding coefficient and multilocus heterozygosity: theoretical expectations and empirical data. Heredity 93:255-265.

Tarvin, K. A., M. S. Webster, E. M. Tuttle, and S. Pruett-Jones. 2005. Genetic similarity of social mates predicts the level of extrapair paternity in splendid fairy-wrens. Anim. Behav. 70:945-955.
